# Computed tomography trachea volumetry in patients with scleroderma: Association with clinical and functional findings

**DOI:** 10.1371/journal.pone.0200754

**Published:** 2018-08-01

**Authors:** Bruno Rangel Antunes Silva, Rosana Souza Rodrigues, Rogério Rufino, Cláudia Henrique Costa, Veronica Silva Vilela, Roger Abramino Levy, Alan Ranieri Medeiros Guimarães, Alysson Roncally Silva Carvalho, Agnaldo José Lopes

**Affiliations:** 1 Postgraduate Programme in Medical Sciences, School of Medical Sciences, State University of Rio de Janeiro, Rio de Janeiro, Brazil; 2 Department of Radiology, Federal University of Rio de Janeiro, Rio de Janeiro, Brazil; 3 Laboratory of Respiration Physiology, Carlos Chagas Filho Institute of Biophysics, Federal University of Rio de Janeiro, Rio de Janeiro, Brazil; 4 Laboratory of Pulmonary Engineering, Biomedical Engineering Programme, Alberto Luiz Coimbra Institute of Post-Graduation and Research in Engineering, Federal University of Rio de Janeiro, Rio de Janeiro, Brazil; Keio University, JAPAN

## Abstract

**Background:**

In scleroderma, excessive collagen production can alter tracheal geometry, and computed tomography (CT) volumetry of this structure may aid in detecting possible abnormalities. The objectives of this study were to quantify the morphological abnormalities in the tracheas of ​​patients with scleroderma and to correlate these findings with data on clinical and pulmonary function.

**Methods:**

This was a cross-sectional study in which 28 adults with scleroderma and 27 controls matched by age, gender and body mass index underwent chest CT with posterior segmentation and skeletonization of the images. In addition, all participants underwent pulmonary function tests and clinical evaluation, including the modified Rodnan skin score (mRSS).

**Results:**

Most patients (71.4%) had interstitial lung disease on CT. Compared to controls, patients with scleroderma showed higher values ​​in the parameters measured by CT trachea volumetry, including area, eccentricity, major diameter, minor diameter, and tortuosity. The tracheal area and equivalent diameter were negatively correlated with the ratio between forced expiratory flow and forced inspiratory flow at 50% of forced vital capacity (FEF_50%_/FIF_50%_) (r = -0.44, p = 0.03 and r = -0.46, p = 0.02, respectively). The tracheal tortuosity was negatively correlated with peak expiratory flow (r = -0.51, p = 0.008). The mRSS showed a positive correlation with eccentricity (r = 0.62, p < 0.001) and tracheal tortuosity (r = 0.51, p = 0.007), while the presence of anti-topoisomerase I antibody (ATA) showed a positive correlation with tracheal tortuosity (r = 0.45, p = 0.03).

**Conclusions:**

In a sample composed predominantly of scleroderma patients with associated interstitial lung disease, there were abnormalities in tracheal geometry, including greater eccentricity, diameter and tortuosity. In these patients, abnormalities in the geometry of the trachea were associated with functional markers of obstruction. In addition, tracheal tortuosity was correlated with cutaneous involvement and the presence of ATA.

## Introduction

Scleroderma or systemic sclerosis (SSc) is a chronic progressive autoimmune disease of connective tissue characterized by microvascular involvement, activation of the immune system, and increased deposition of extracellular matrix in the skin and internal organs by excess collagen fibers, leading to fibrosis [1–4]. Scleroderma is eight times more common in females than in males; its reported prevalence is approximately 10 cases per 100,000 person, and this rate is probably underestimated [5]. The disease can affect several organs and systems; the skin is the most frequently affected site, followed by the lungs, kidneys, musculoskeletal system, cardiovascular system, and gastrointestinal tract. The presence of multiple affected sites worsens the prognosis [1].

In scleroderma, thoracic involvement is observed mainly as diffuse fibrosis or pulmonary hypertension; these conditions are associated with limited (lc-SSc) and diffuse cutaneous (dc-SSc) forms of scleroderma, respectively [1]. In an autopsy study, parenchymal involvement was seen in up to 100% of patients with scleroderma [2]. Although interstitial lung involvement is subclinical and asymptomatic at early stages in most patients, interstitial lung disease (ILD) associated with scleroderma (ILD-SSc) is observed in approximately 40% of cases and is a major cause of morbidity and mortality [6]. Despite the constellation of thoracic manifestations that occur in patients with scleroderma, little is known about the involvement of the trachea in these patients. Ooi et al. [7] reported that scleroderma affected small and large airways in 45–100% of patients. In this context, the trachea has been a 'forgotten zone' in the study of several diseases because the pathological processes involving this structure have often not received the necessary clinical recognition.

Computed tomography (CT) has become an important part of the detection and evaluation of routine thoracic involvement in scleroderma, and the abnormalities observed by this method are closely correlated with the observed physiological parameters [2]. In the last two decades, various computer tools that can be used to automatically slice the chest using CT images have been developed. These include multiplanar reformation, regional lung attenuation analysis of lung tissue and quantification of anatomical images, including the area and volume of airways and lungs [2,7]. In scleroderma, computer-assisted tomography analysis is highly efficient and, in combination with physiological and patient-centered measurements, may provide a means to accurately assess and monitor lung disease progression and response to therapy [2,8,9].

An understanding of the acquisition, processing and analysis of CT scans and how these processes affect the imaging of the trachea is essential for assessing the accuracy of the measurements and making effective use of newly available tools. In the study of the trachea, the cross-sectional area and diameter are the most commonly measured dimensions; in the context of assessing possible obstruction, length and caliber are important [10–13]. More recently, the process of skeletonization and volumetry of the airways through CT in normal individuals and in individuals with some clinical conditions has been described [10–15]. In this method, the digital imaging component is transformed into a subset of the original component [10]. After automated segmentation of CT images, the skeletonization algorithm allows the extraction of a tracheal centerline and facilitates the reconstruction of CT data orthogonal to the reduction of the effects of partial volume averages [11]. However, no study has explored the use of this resource in the evaluation of the tracheas of patients with scleroderma.

We hypothesized that the excessive production of collagen that occurs in scleroderma alters the geometry of the trachea and that the skeletonization of this structure would aid in the detection of possible abnormalities. Thus, the present study aimed to identify and quantify the morphological abnormalities in the tracheas of ​​patients with scleroderma and, secondarily, to correlate these findings with data on clinical and pulmonary function.

## Methods

### Patients

This cross-sectional study was conducted between February 2016 and September 2017 in 43 consecutive patients with scleroderma who were ≥18 years of age, of both sexes, and were seen regularly at the Piquet Carneiro Polyclinic of the State University of Rio de Janeiro, Rio de Janeiro, Brazil. The patients included in the study had been diagnosed with scleroderma by a rheumatologist according to the parameters set forth by the American College of Rheumatology/European League Against Rheumatism [1]. The following exclusion criteria were used: clinical instability; history of respiratory infection in the last three weeks; history of previous or current smoking; evidence of overlapping of scleroderma with other connective tissue diseases; report of previous tracheal or pleuropulmonary disease not related to scleroderma; and inability to perform pulmonary function tests (PFTs). Regarding cutaneous involvement, patients were classified as having lc-SSc (thickening of the skin distal to the elbows and knees and proximal to the clavicles, including the face) or dc-SSc (thickening of the proximal skin as well as of the skin distal to the elbows and knees and including the trunk and face) [16]. The modified Rodnan skin score (mRSS) was used to assess skin damage in patients with scleroderma. In this system, a score of 0 (no thickening), 1 (light thickening), 2 (moderate thickening) or 3 (severe thickening) is assigned to each area, resulting in a total score ranging from 0 (best) to 51 (worst) [17]. The presence of autoantibodies, including anti-topoisomerase I and anti-centromere, was also investigated.

We also evaluated a control group of 27 individuals aged ≥ 18 years of both sexes. The subjects in the control group were asked to perform the PFTs after undergoing chest CT scanning in our service for the following reasons: investigation of contact with tuberculosis patients (n = 9); staging of neoplasms outside the thorax (n = 7); evaluation of trauma (n = 6); and evaluation of fever of unknown origin (n = 5). The following criteria were used to select subjects for the control group: no previous history of smoking or chronic tracheal or pleuropulmonary diseases; chest CT scans without abnormalities; and lung function parameters within the normal range (*i*.*e*., no value below the lower limit of normal or above the upper limit of normal in relation to the predicted value). All CT scans and PFTs for the control group were performed using the same equipment that was used for the scleroderma group.

The protocol was approved by the Research Ethics Committee of the State University of Rio de Janeiro under the number CAAE- 50752615.9.0000.5259, and it complied with the current national and international standards. All individuals signed an informed consent form.

### Pulmonary function testing

The PFTs performed were spirometry, body plethysmography, and diffusion capacity for carbon monoxide (DLco). The exams were performed on an HDpft 3000 (nSpire Health, Inc., Longmont, CO, USA) according to the standards set forth by the American Thoracic Society [18]. The Brazilian reference values [19,20] were used, and the results are expressed as percentages of the predicted values. An increase in the ratio between forced expiratory flow and forced inspiratory flow at 50% of forced vital capacity-FVC (FEF_50%_/FIF_50%_) > 1.50 was used as an indicator of extrathoracic airway obstruction [21].

### CT scan interpretation and protocol

CT scans were performed on a 64-channel multislice Brilliance 40 scanner (Philips Medical Systems, Cleveland, OH, USA) that was capable of performing volumetric acquisitions with subsequent multiplanar reconstructions. The acquisitions were performed in the axial plane with patients in the supine position using the technical parameters 120 kV and 458 mA (these parameters varied according to the biotype of the patient), slice thickness 2 mm, and pitch 2 mm from the jugular notch to the xiphoid process in maximal inspiration and expiration. After acquisition of the images, a high-resolution reconstruction with a matrix of 512 × 512 was performed using a high-frequency algorithm, a window width of 1200 HU, and a level centered at -800 HU.

Parenchymal abnormalities on CT were interpreted by two independent readers (R.S.R. and G.B.C.) who were blinded to patient history and physiological results; a consensus opinion was reached in cases in which there was disagreement. CT scans were reviewed at five levels, as follows: 1) origin of major vessels; 2) carina; 3) confluence of pulmonary veins; 4) halfway between the third and the fifth sections; 5) 1 cm above the right hemidiaphragm [22,23]. The total extent of ILD was estimated to the nearest five percent in each of the five levels, with global extent of disease on CT as the mean of the scores [22–24]. The coarseness of pulmonary fibrosis was evaluated as follows: 0, ground-glass opacification alone; 1, fine intralobular fibrosis; 2, microcystic honeycombing comprising air spaces ≤ 4 mm in diameter; and 3, macrocystic honeycombing comprising air spaces > 4 mm in diameter. The total coarseness score for each patient was derived by summing the scores at the five levels (range 0 to 15) [22,23]. For each patient, the total extension of ILD and the coarseness of pulmonary fibrosis were derived by averaging the scores at each level assessed by the two independent readers. Finally, the extent of ILD-SSc was classified as limited (lung parenchyma involvement <20%) or extensive (>20%). For indeterminate cases, ILD-SSc was considered extensive if FVC <70% and limited if FVC >70% [22,25].

### Imaging processing

The airways were segmented using 3DSlicer version 4.4.0 (http://slicer.org) [26] with the aid of its AirwaySegmentation extension. At the end of processing, a nearly raw raster data (.nrrd) file was saved. Using AirwayProcessing, two distinct processes were executed: 1) the .nrrd file was read and transformed into a binary matrix of data; in this way, the process of skeletonization was initiated, and a skeleton composed of several points was then produced; 2) using the skeleton and its coordinates (X, Y and Z axes), cross-sectional planes were generated. Individual processing of all points was performed using the slice command, and the processing angles were defined by a normal line between the point in question and the next five points. The plane of square cross-section was formed by a square grid with 70 pixels on the side. Using MATLAB 2014a (MathWorks Inc., Natick, MA, USA), information obtained individually by the slice command was catalogued using the ‘regionprops’ command.

**Fig 1:**
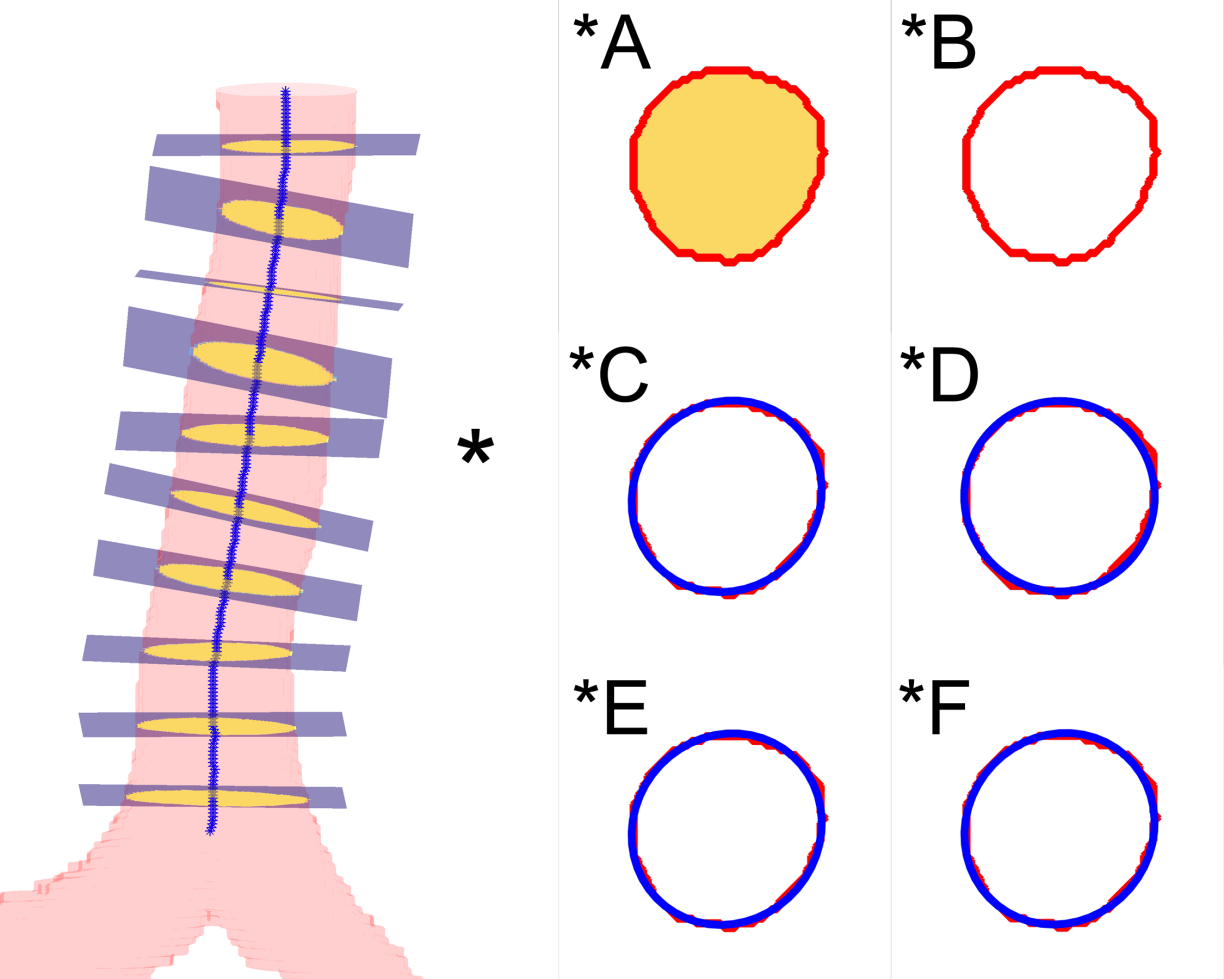
3D reconstruction, cross-sectional planes, skeleton and indication of the plane used for representation. The letters A to F and the values ​​for the selected plane indicate, respectively: (*A) area (182.4 mm^2^), which is the product of the pixel size and the number of pixels in the region (in this figure, the area is shown in yellow and includes the red line that demarcates the perimeter); (*B) perimeter (47.3 mm), which is the length of the line indicated in red; (*C) eccentricity (0.35), which is indicated by the equivalent ellipse drawn in blue; (*D) equivalent diameter (15.2 mm), which is the circle of area equivalent to the cross-sectional area; (*E) major diameter (15.8 mm), which is the longest segment of the equivalent ellipse (indicated by the inner line); and (*F) minor diameter (14.8 mm), which is the smallest segment (indicated by the inner line).

Fig 1 shows the 3D reconstruction, the tracheal skeleton, the cross-sectional planes and their geometric parameters [9,10]. The scheme show in Fig 1 was chosen for illustrative purposes and accurately represents the region of the trachea in a 2D environment. In Fig 1, the axial sections indicated by specific letters represent area (*A), perimeter (*B), eccentricity (*C), equivalent diameter (*D), major diameter (*E) and minor diameter (*F). It is emphasized that the segments shown in red and blue in Fig 1 were enlarged for visualization purposes.

Thus, the following measures were calculated for each study participant:

1) Area: the measured area of the tracheal lumen intersected by the cross-sectional plane. The area was calculated by multiplying the pixel area value by the number of pixels present, as shown in Eq 1,

Area=ps*n (1)

where *ps* is the individual area of ​​each pixel and *n* is the number of pixels.

2) Perimeter: the length of the outer margin of the tracheal lumen.
3) Eccentricity: a parameter associated with the ellipsoidal shape of a given region. It is the ratio of the focus of the ellipse to its largest diameter. The result is a value between 0 and 1, where 0 is the representation of a circle and 1 is the representation of a line. Fig 1*C illustrates eccentricity as the equivalent ellipse (shown in blue); the outer edge of the tracheal lumen is shown in red. The eccentricity was calculated according to Eq 2,

$$Eccentricity=2*\frac{\sqrt{{\frac{\left(MaAL\right)}{2}}^{2}-\frac{{\left(MiAL\right)}^{2}}{2}}}{MaAL}$$ (2)

where *MaAL* represents the largest diameter and *MiAL* the smallest diameter of the ellipse.

4) Equivalent diameter: a circle of the same area as ​​the region intersected by the cross-sectional plane. In Fig 1*D, the equivalent diameter is shown as a blue circle with an area equivalent to that of the tracheal lumen in the same region. The equivalent diameter was calculated using Eq 3,

$$Equivalent\;diameter=\frac{\sqrt{4*area}}{\pi }$$ (3)

5) Major diameter: the length of the longest segment of the ellipse equivalent to the region. Similarly, the minor diameter represents the length of the smallest segment of the ellipse. Fig 1*E and 1*F denote the perimeter of the tracheal lumen (red) along with its ellipse (blue); the center lines indicate the largest and the smallest segment, respectively, of each region.

**Fig 2:**
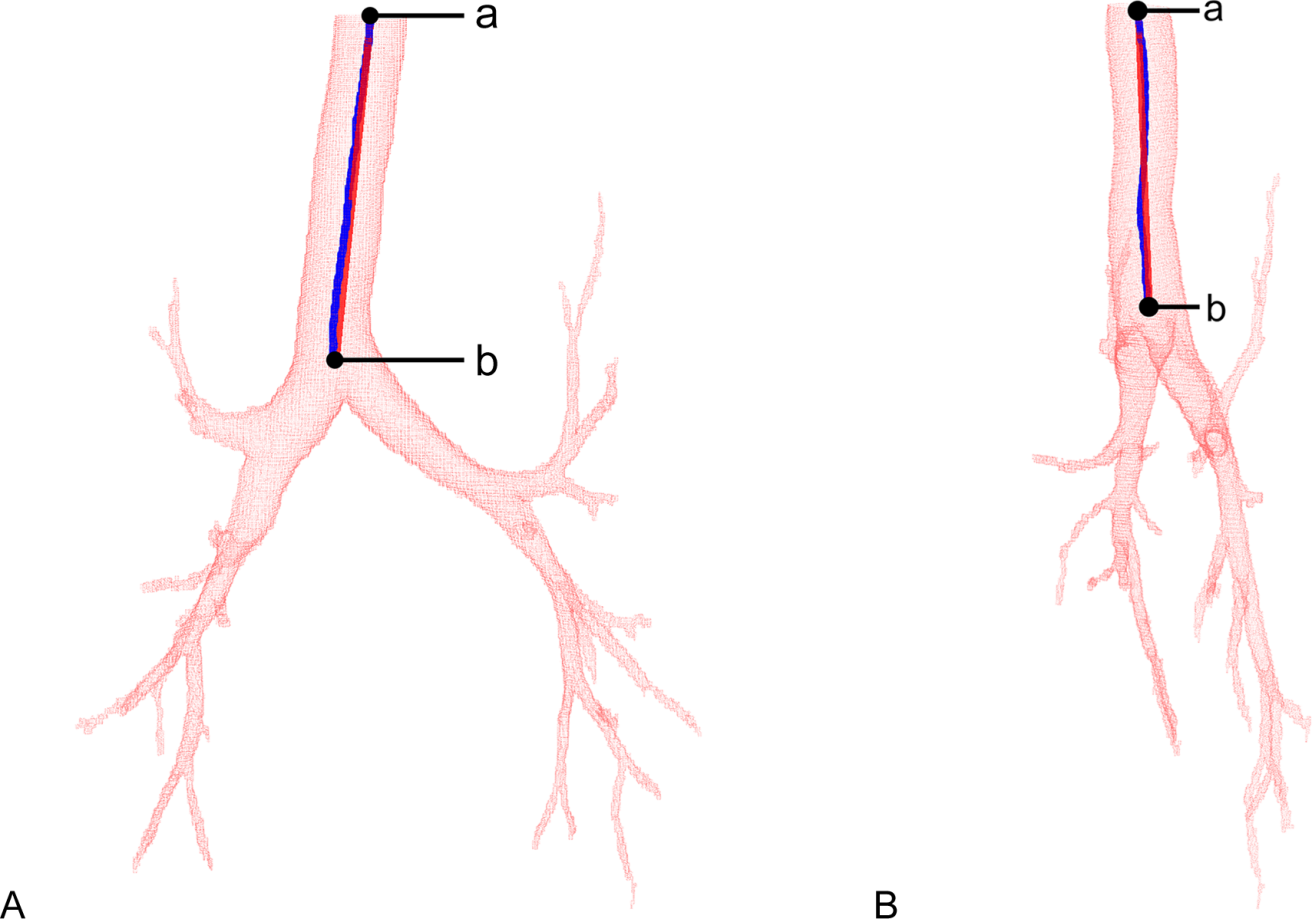
Measurement of the tortuosity of the trachea. Images in the coronal (A) and sagittal (B) planes are shown. In this scheme, *L* is the length of the trachea (the total length of 'ab', shown in blue) and *vd* is the vectorial distance between the points at the extremities (the length of the shortest possible path between 'a’ and ‘b', shown in red).

We also calculated the tortuosity (sinuosity index), which is the deviation of the central axis of the trachea considering its proximal and distal extremities. Mathematically, the tortuosity was determined from the sum of the Euclidean distance of each segment of the trachea, with a distance equivalent to the thickness of each cross-section of the original CT image, divided by the Euclidean distance of the end points of the trachea. The tortuosity may be expressed succinctly using the formula described in Eq 4,

$$Tortuosity=\frac{L}{vd}$$ (4)

where *L* is the length of the trachea and *vd* is the vectorial distance between the points at the extremities (Fig 2). The tortuosity is expressed as a value ≥ 1; the higher the index, the greater the tortuosity of the trachea [27].

**Fig 3:**
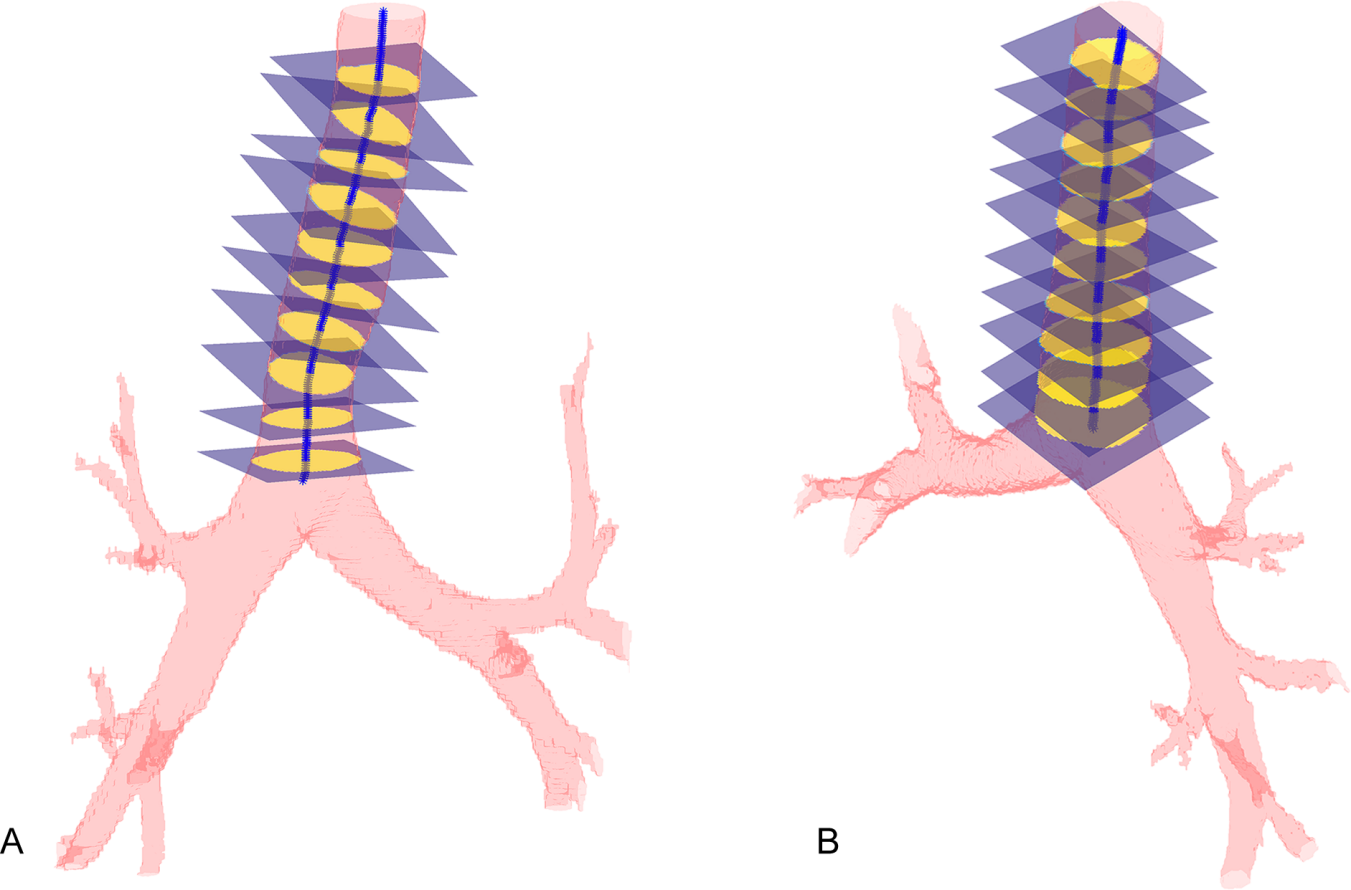
Skeletonization process of the trachea. Three-dimensional reconstruction of the trachea of one study participant in two planes (A and B). The median values showed in the ten planes of square cross-sections are as follows: area = 184.5 mm^2^; perimeter = 47.6 mm; eccentricity = 0.51; equivalent diameter = 15.3 mm; major diameter = 15.9 mm; minor diameter = 14.4 mm; and tortuosity = 1.027.

Fig 3 exemplifies the skeletonization process of the trachea of a study participant. The figure shows the three-dimensional reconstruction images of the trachea in two different planes.

There is inherent variability in the measures used to determine the quantitative metrics of eccentricity and tortuosity. For eccentricity, there is a value for each section plane of the trachea of a given individual (Fig 3). We use the median to represent the value of each individual; therefore, there are *n* medians, where *n* is the number of elements in each group of variables. For tortuosity, only one value was calculated for each trachea; it depends on the extreme values (proximal and distal), as shown in Fig 2.

### Statistical analysis

To better evaluate the effect of the extent of ILD-SSc on the clinical variables, PFTs, and CT trachea volumetric parameters, we divided the participants into three subgroups as follows: 1) a control group; 2) a scleroderma group with no pulmonary involvement/limited ILD; and 3) a scleroderma group with extensive ILD. ANOVA followed by the Bonferroni post hoc test was used to compare the results obtained for the patients in these three groups. Comparison of the clinical variables, PFTs, and CT trachea volumetric parameters of the SSc patients without pulmonary involvement with those of control subjects and with those of SSc patients with no pulmonary involvement/limited ILD-SSc and SSc patients with extensive ILD-SSc was performed using Student's t-test for independent samples in the case of numerical data and using the chi-square or Fisher's exact test in the case of categorical data. To evaluate the associations between the numerical variables of PFTs and CT trachea volumetric parameters, the Pearson correlation coefficient (r) was used. The criterion for determining significance was 5%. Statistical analysis was performed using SAS 6.11 software (SAS Institute, Inc., Cary, NC, USA).

## Results

Of the 43 patients included in the study, 15 were excluded for the following reasons: reported being a smoker (n = 8); history of previous pleuropulmonary disease not associated with scleroderma (n = 3); inability to perform PFTs (n = 2); scleroderma-polymyositis overlap syndrome (n = 1); and scleroderma-rheumatoid arthritis overlap syndrome (n = 1). In the total sample of patients, the mean age of subjects with scleroderma was 52.5 ± 11 years, and 25 (89.3%) were women. The disease duration was 4.21 ± 2.50 years from the onset of non-Raynaud’s phenomenon and 9.65 ± 5.13 years from the onset of Raynaud’s phenomenon symptoms. Nineteen patients (67.9%) had lc-SSc, and nine (32.1%) had dc-SSc; of the latter, six (21.4%) had a mRSS > 18. Anti-topoisomerase I antibody (ATA), anticentromere antibody and anti-RNA polymerase III were positive in 13 (46.4%), six (21.4%) and three (10.7) patients, respectively; no autoantibodies were identified in six (21.4%) patients. Seven patients (25%) had an FEF_50%_/FIF_50%_ ratio > 1.50. Comparisons of clinical data, pulmonary function parameters and computed tomography scores in the control group, the scleroderma group with no pulmonary involvement/limited ILD, and the scleroderma group with extensive ILD are shown in Table 1. In addition, comparisons of lung function parameters in the control group and in patients without pulmonary involvement on CT did not show any significant difference (p > 0.05 for all).

**Table 1:** Demographic characteristics, clinical data, pulmonary function and computed tomography scores of patients with scleroderma and of patients in the control group.

Variable	Control group (n = 27)	Scleroderma group with no pulmonary involvement/limited ILD (n = 16)	Scleroderma group with extensive ILD (n = 12)	p value
Demographic data				
Females	23 (85.2)	14 (87.5)	11 (91.7)	0.41
Age (years)	49 ± 12.6	51.7 ± 10	52.8 ± 11.2	0.28
BMI (kg/m^2^)	27.2 ± 5.87	24.2 ± 5.22	25.1 ± 5.30	0.09
Type of scleroderma				
lc-SSc	-	12 (75)	7 (58.3)	0.34
dl-SSc	-	4 (25)	5 (41.7)	
mRSS	-	10.3 ± 7.44	11.8 ± 7.60	0.12
Type of autoantibody				
Anti-topoisomerase I antibody	-	5 (31.2)	8 (66.7)	0.28
Anticentromere antibody	-	5 (31.2)	1 (8.33)	
Anti-RNA polymerase III	-	2 (12.5)	1 (8.33)	
Autoantibody not identified	-	4 (25)	2 (16.7)	
Lung function				
FVC (L)	3.08 ± 0.81	2.62 ± 0.72*	2.25 ± 0.87*†	**0.008**
FVC (% predicted)	102.4 ± 19.8	85 ± 19.3*	68 ± 21.5*†	**0.005**
FEV_1_ (L)	2.51 ± 0.75	2.11 ± 0.63*	1.80 ± 0.59*†	**0.009**
FEV_1_ (% predicted)	100.7 ± 18.5	83.1 ± 16*	69.2 ± 17.8*†	**0.006**
FEV_1_/FVC (%)	78 ± 12.6	80 ± 12	86 ± 11.3	0.11
PEF (L/s)	7.75 ± 2.14	6.25 ± 2.11	5.26 ± 1.97*	**0.032**
PEF (% predicted)	110 ± 31.7	86 ± 30.5	75.5 ± 29.2*	**0.025**
FEF_50%_/FIF_50%_ (%)	1.04 ± 0.62	1.28 ± 0.72*	1.34 ± 0.70*	**0.046**
DLco (mL/min/mmHg)	20.6 ± 3.44	18 ± 4.22*	9.27 ± 3.23*†	**0.003**
DLco (% predicted)	97 ± 21.6	70.3 ± 18.3*	60 ± 17.8*†	**0.001**
Raw (cm H_2_O/L/s)	1.58 ± 0.75	1.78 ± 0.77	1.93 ± 0.78	0.32
SGaw (L/s/cm H_2_O/L)	0.210 ± 0.078	0.245 ± 0.089	0.257 ± 0.103	0.09
Computed tomography scores				
ILD-SSc extent (% parenchyma)		9.63 ± 8.80	28.1 ± 10.4	**< 0.0001**
Coarseness of pulmonary fibrosis		2.15 ± 1.44	6.35 ± 4.20	**< 0.0001**

The values shown are means ± SD or number (%). Bold type indicates significant differences.

*Significantly different from control group.

†Significantly different from scleroderma group with no pulmonary involvement/limited ILD-SSc. ILD-SSc = interstitial lung disease associated with scleroderma. BMI = body mass index; lc-SSc = limited cutaneous form; dc-SSc = diffuse cutaneous form; mRSS = modified Rodnan skin score; FVC = forced vital capacity; FEV_1_ = forced expiratory volume in one second; PEF = peak expiratory flow; FEF_50%_/FIF_50%_ = ratio between the forced expiratory flow and forced inspiratory flow at 50% of forced vital capacity; DLco = diffusing capacity for carbon monoxide; Raw: airway resistance; SGaw: specific airway conductance.

On thorax CT, 16 patients (57.1%) were classified as having no pulmonary involvement (n = 8) or limited pulmonary involvement (n = 8), while 12 (42.9%) were classified as having extensive pulmonary involvement. In the measurements obtained through CT trachea volumetry, patients with scleroderma presented higher values ​​for the following parameters: area, eccentricity, major diameter, minor diameter, and tortuosity. Table 2 compares the CT trachea volumetry findings for control subjects, patients in the scleroderma group with no pulmonary involvement/limited ILD, and patients in the scleroderma group with extensive ILD.

**Table 2:** Variables of CT trachea volumetry according to group.

Variable	Control group (n = 27)	Scleroderma group with no pulmonary involvement/limited ILD (n = 16)	Scleroderma group with extensive ILD (n = 12)	p value
Area (mm^2^)	207.6 ± 32.6	211.3 ± 38.2*	214.6 ± 43.6*	**0.028**
Perimeter (mm)	45.3 ± 4.12	49.2 ± 4.45	51.5 ± 4.67	0.12
Eccentricity	0.46 ± 0.04	0.51 ± 0.06*	0.54 ± 0.06*	**0.013**
Equivalent diameter (mm)	16.1 ± 1.24	16.2 ± 1.55	16.5 ± 1.60	0.82
Major diameter (mm)	17.1 ± 2.22	17.6 ± 1.92*	18.2 ± 2.01*	**0.039**
Minor diameter (mm)	14.5 ± 2.11	15 ± 1.72*	15.3 ± 1.85*	**0.043**
Tortuosity	1.023 ± 0.014	1.039 ± 0.014*	1.058 ± 0.015*†	**0.009**

The values shown are means ± SD or number (%). Bold type indicates significant differences.

*Significantly different from control group.

†Significantly different from scleroderma group with no pulmonary involvement/limited ILD-SSc. ILD-SSc = interstitial lung disease associated with scleroderma.

We also compared patients without pulmonary involvement on CT (n = 8) with controls. In this evaluation, patients without pulmonary involvement on CT showed higher values, with significant differences in the following CT tracheal volumetric findings: area (210.7 ± 35 vs. 207.6 ± 32.6 mm^2^, p = 0.031; eccentricity (0.50 ± 0.05 vs. 0.46 ± 0.04, p = 0.018); major diameter (17.5 ± 1.70 vs. 17.1 ± 2.22 mm, p = 0.039); minor diameter (14.9 ± 1.68 vs. 14.5 ± 2.11 mm, p = 0.044); and tortuosity (1.039 ± 0.017 vs. 1.023 ± 0.014, p = 0.01).

**Fig 4:**
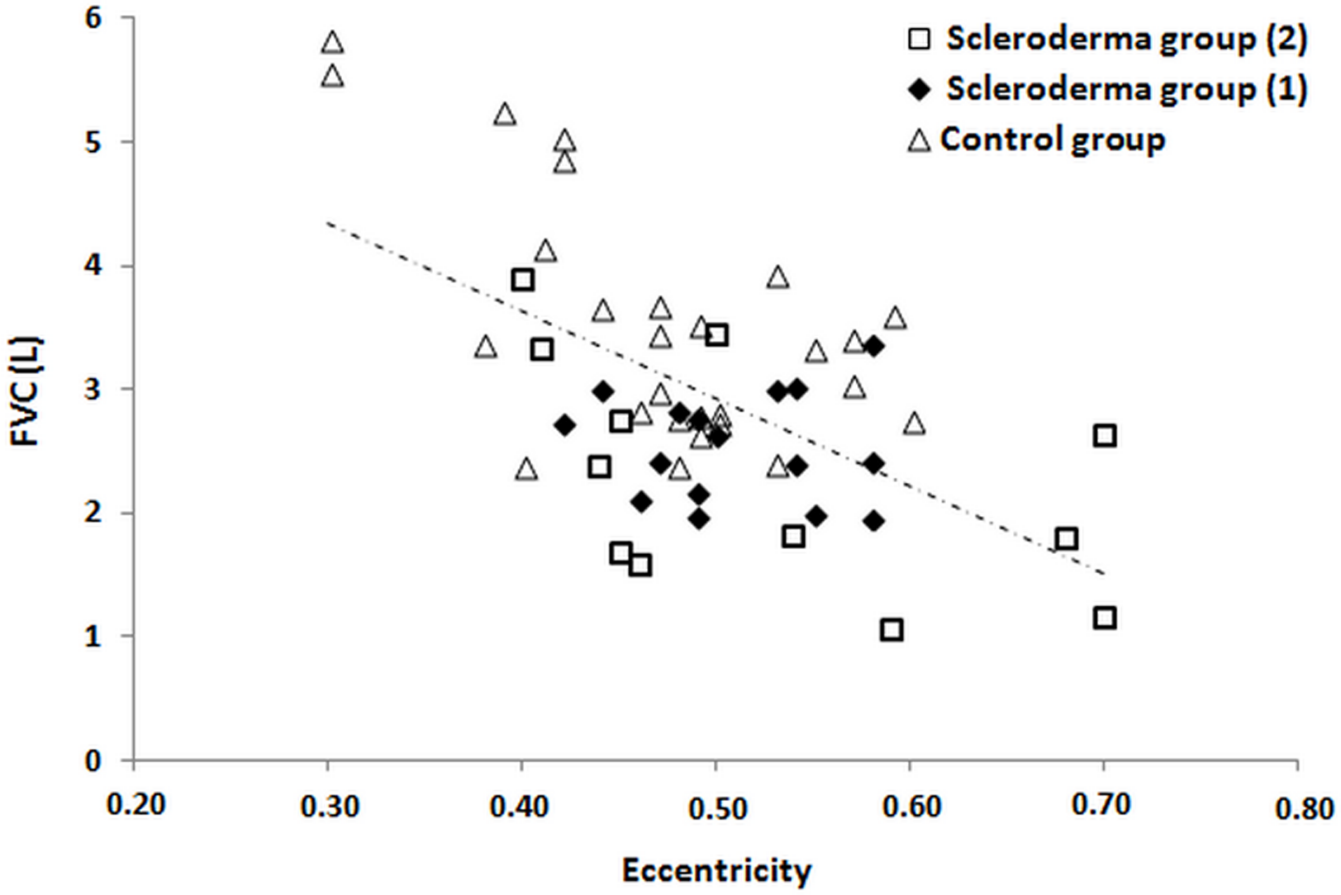
Relationship between forced vital capacity (FVC) and eccentricity of the trachea (r = -0.57, p = 0.002). Scleroderma group (1) = scleroderma group with no pulmonary involvement/limited interstitial lung disease; scleroderma group (2) = scleroderma group with extensive interstitial lung disease.

**Fig 5:**
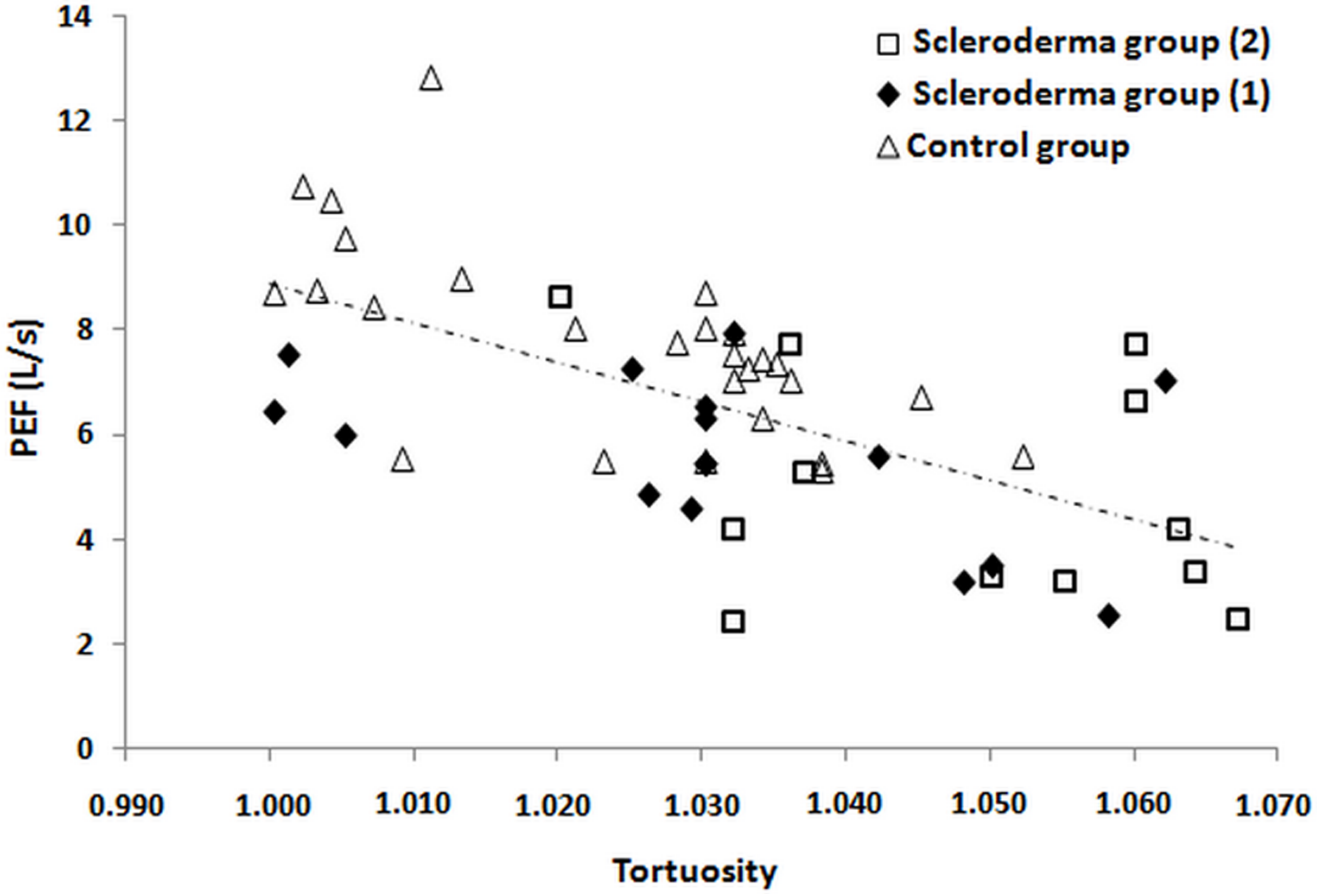
Relationship between peak expiratory flow (PEF) and tortuosity of the trachea (r = -0.51, p = 0.008). Scleroderma group (1) = scleroderma group with no pulmonary involvement/limited interstitial lung disease; scleroderma group (2) = scleroderma group with extensive interstitial lung disease.

We evaluated the correlations between the parameters provided by CT trachea volumetry and the pulmonary function indices (absolute values) (Table 3). The tracheal area was negatively correlated with FEF_50%_/FIF_50%_ (r = -0.44, p = 0.03), while the eccentricity was negatively correlated with FVC (r = -0.57, p = 0.002) (Fig 4) and forced expiratory volume in one second (r = -0.50, p = 0.009). The equivalent diameter was negatively correlated with FEF_50%_/FIF_50%_ (r = -0.46, p = 0.02), while tortuosity was negatively correlated with peak expiratory flow (PEF) (r = -0.51, p = 0.008) (Fig 5). A positive correlation of tortuosity with coarseness of pulmonary fibrosis in CT was also found (r = 0.45, p = 0.02).

**Table 3:** Pearson’s correlation coefficients for CT trachea volumetry, pulmonary fibrosis and pulmonary function.

	FVC	FEV_1_	FEV_1_/FVC	PEF	FEF_50%_/FIF_50%_	DLco	Raw	SGaw	Coarseness of fibrosis
Area	-0.13	-0.11	-0.06	0.24	-0.44*	-0.24	0.12	-0.19	0.25
Perimeter	-0.09	-0.07	-0.08	0.25	**-0.47†**	-0.18	0.13	-0.21	0.17
Eccentricity	**-0.57**‡	**-0.50**†	-0.12	-0.07	-0.17	-0.35	-0.11	-0.04	0.26
Equivalent diameter	-0.11	-0.10	-0.07	0.25	**-0.46**†	-0.27	0.11	-0.21	0.18
Major diameter	-0.04	-0.06	-0.08	0.25	-0.42*	-0.16	0.07	-0.20	0.22
Minor diameter	-0.22	-0.19	-0.03	0.21	**-0.47**†	-0.11	0.06	-0.17	0.25
Tortuosity	-0.17	-0.15	-0.26	**-0.51**†	-0.28	-0.12	0.09	-0.15	0.45*

FVC = forced vital capacity; FEV_1_ = forced expiratory volume in one second; PEF = peak expiratory flow; FEF_50%_/FIF_50%_ = ratio between the forced expiratory flow and forced inspiratory flow at 50% of forced vital capacity; DLco = diffusing capacity for carbon monoxide; Raw = airway resistance; SGaw = specific airway conductance. Bold type indicates significant differences.

*p < 0.05

†p < 0.01

‡p < 0.005

We also evaluated the correlations between the clinical findings and the parameters provided by CT trachea volumetry. The mRSS showed a positive correlation with eccentricity (r = 0.62, p < 0.001) and tortuosity (r = 0.51, p = 0.007). In turn, the presence of anti-topoisomerase I antibody showed a positive correlation with tortuosity (r = 0.45, p = 0.03).

## Discussion

The main findings of the present study were that in a sample composed predominantly of scleroderma patients with associated ILD, the trachea showed greater area, eccentricity, and tortuosity. In these patients, a greater tracheal tortuosity led to a smaller airflow; in addition, the lower the tracheal diameter, the greater the degree of airway obstruction. Furthermore, tracheal tortuosity was associated with the presence of pulmonary fibrosis, the presence of ATA, and greater mRSS. To our knowledge, this is the first study to show abnormalities in the structure and function of the trachea as well as its correlations with clinical findings in patients with scleroderma.

In recent years, several CT image enhancement techniques have been developed in an attempt to find a method that is both quantitative and reliable and allows more accurate assessment than conventional visual reading. Compared to the traditional visual interpretation of CT findings, computer-based automatic evaluation can improve the objectivity, sensitivity, and repeatability of quantitative chest imaging analyses [2,10]. In this study, we applied a computer-assisted method to evaluate the tracheas of ​​patients with scleroderma in an unprecedented way considering that computerized evaluation has previously only been used in the study of ILD-SSc [2,28]. Our results indicate that CT trachea volumetry and subsequent skeletonization of the images provide a number of interesting measures for the best evaluation of patients with scleroderma. We observed that patients with scleroderma presented greater area, eccentricity, and tortuosity of the trachea. In scleroderma, myofibroblasts constitutively secrete components of the extracellular matrix and exert excessive cicatrization of the skin and internal organs [3,29]. Thus, we think that the cicatricial changes that occur at the level of the neck can directly impact the geometry of the trachea and alter the measures of diameter and tortuosity of this structure.

In our study, the deviations of the trachea were measured by means of the tortuosity (sinuosity index) after the skeletonization process. The skeletonization process has been used previously by us to obtain the skeletons of specific structures by thinning [10]. Interestingly, we found an association between tracheal tortuosity and the pulmonary fibrosis score obtained by semi-quantitative CT reading. This association between structural alterations of the trachea and the lung, despite being evaluated by different techniques in our study, suggests that the deformity of the trachea in scleroderma may be an extension of the fibrotic disease that occurs at the pulmonary level. However, it is worth mentioning that in our study, patients without pulmonary involvement showed higher values in tracheal volumetry than control subjects. This finding suggests that factors other than pulmonary fibrosis are involved in the tracheal abnormalities that occur in SSc patients. Consistent with this observation, several studies have shown impairment of anatomic structures above the trachea in scleroderma patients; laryngeal dysfunction is relatively common, with pathologic findings demonstrating fibrinoid degeneration and an increase in collagen fibers [30,31]. Considering the promising advent of user-friendly software, evaluation of the trachea in patients with scleroderma may be an interesting approach both in clinical practice and in trials. Thus, precise characterization of the trachea in scleroderma may offer an additional tool for the follow-up of these patients and may be helpful in the evaluation of clinical treatment.

The presence of obstructive abnormalities in the cervical trachea can be detected by the flow-volume loop of spirometry even when there is no clinical suspicion, with the most used index being the ratio FEF_50%_/FIF_50%_ [32]. In the present study, 25% of our patients had an FEF_50%_/FIF_50%_ > 1.50, and we found negative correlations between FEF_50%_/FIF_50%_ and several indices measured by CT trachea volumetry, including area, perimeter, equivalent diameter, major diameter, and minor diameter. Consistent with our findings, Miranda et al. [33] used the forced oscillation technique in patients with scleroderma and found an increase in mean resistance, a parameter that reflects changes in the most central airways. Another study using impulse oscillometry observed an increase in the resistive and reactive properties of the respiratory system in patients with scleroderma, and these alterations were correlated with the findings of fibrosis in CT [34]. Interestingly, we observed an association between tracheal tortuosity and PEF (which reflects flow through large airways), supporting the notion that tracheal deviation is a key contributor to the reduction of airflow in the large airways of patients with scleroderma. However, quantitative measurement of eccentricity was not correlated with FEF_50%_/FIF_50%_. One possible explanation for this finding is that increased tracheal area in scleroderma patients may at least partially counterbalance the effects of reduced inspiratory flow in these patients.

In our study, patients with extensive ILD displayed greater tracheal area than patients without pulmonary involvement and patients with limited ILD; all of these patients, in turn, displayed greater tracheal area than the control subjects. Several recent studies have shown that larger esophageal diameter is associated with higher pulmonary fibrosis and worse lung function in individuals with scleroderma [35,36]. Since microaspiration secondary to gastro-esophageal reflux is associated with the progression of pulmonary fibrosis in scleroderma [37,38], we think that the tracheal pathology may also contribute to this phenomenon. Longitudinal and controlled studies of larger numbers of patients are required to evaluate whether or not tracheal pathology is involved in the progression of lung fibrosis in patients with scleroderma.

In the present study, the mean value of FVC was higher in the control group and lower in the scleroderma group with extensive ILD and showed an intermediate value in the scleroderma group with no pulmonary involvement (or limited ILD) (102.4 ± 19.8% vs. 85 ± 19.3% vs. 68 ± 21.5% predicted, p = 0.005). Although the restrictive pattern in scleroderma is largely explained by the presence of ILD, a reduced compliance of the respiratory system may also be due to chest wall tightening from skin thickening, pleural disease, cardiac involvement, and respiratory muscle weakness [39–41]. Since no significant difference was observed in FVC between the control group and patients without pulmonary involvement in CT, we believe that ILD is the main contributor to the restrictive damage in our sample. However, we observed a significant correlation between FVC and the quantitative measurement of tracheal eccentricity. A possible explanation for this association is that overproduction of collagen and deposition of connective tissue, which are primary pathophysiological mechanisms of SSc, cause both abnormal tracheal geometry and reduced lung volume in these patients.

In scleroderma, cutaneous involvement occurs due to the increased thickness and hardness of the skin, which leads to shrinking of the skin in deeper structures; although cutaneous involvement is usually most prominent on the face and hands, these abnormalities may extend to the upper chest [42,43]. Some studies have shown that subclinical involvement of the upper chest is detectable by high-frequency ultrasound even with normal palpation [44,45]. In our study, the mRSS (a key measure in the clinical evaluation of patients with scleroderma) was associated with the eccentricity values provided by CT trachea volumetry. Since eccentricity measures the deviation of a conical structure in relation to its circumference [46], we think that skin thickening that occurs at the neck level may negatively impact the geometry of the trachea in patients with scleroderma [47]. Consistent with our findings, Kim et al. [48] found an association between the fibrosis score detected by CT using a computer-assisted method and the mRSS of patients with scleroderma both at baseline and during a 12-month follow-up period. This reinforces the idea that the measure of eccentricity may be a marker of the severity of the process of collagen hyalinization and abnormalities in the elastic tissues that surround the neck and may extend to the intrathoracic structures of patients with scleroderma [42]. Considering that future directions for the management of patients with scleroderma, including epigenetic modulation and antifibrotic or biological therapy, are being discussed [49,50], we think that the parameters provided by the measurement of tracheal geometry can contribute to the evaluation of the outcomes.

The identification of reliable and consistent biomarkers that can be used to predict the course of specific diseases is necessary for better stratification and management of patients and would be of great use in the treatment of a multifaceted disease such as scleroderma [3]. In this context, several attempts have been made in recent years to correlate possible serum biomarkers with clinical features distinct from scleroderma. Several investigators have shown an association between ATA and the risk of development and progression of pulmonary fibrosis in patients with scleroderma [51,52]. A recent study has shown that topoisomerase I peptide-loaded dendritic cells induce not only the autoantibody response but also cutaneous and pulmonary fibrosis [52]. In the present study, we observed a positive correlation between the presence of ATA and tracheal tortuosity. This significant correlation must be viewed cautiously since we did not evaluate predicting values. However, we think that this finding could serve as a starting point for longitudinal studies designed to evaluate a possible contribution of ATA to the management of tracheal pathology in patients with scleroderma.

A critical analysis of the limitations of the present study is pertinent. First, the sample size is small, and the sample is representative of only one center. Second, the sample is composed predominantly of scleroderma patients with associated ILD. Although we observed significant differences in the abnormalities of trachea volumetry between control subjects and patients without pulmonary involvement on CT, a greater number of patients in this group could allow for more robust conclusions. Third, CT pulmonary densitovolumetry could have aided in the study of correlations between tracheal changes and those observed in the lungs and lower airways. Fourth, an assessment comparing patients with ILD-SSc with patients with ILD due to other causes (e.g., idiopathic pulmonary fibrosis, rheumatoid arthritis-ILD) might better define the role of scleroderma in the development of tracheal abnormalities. Finally, the evaluation of other biomarkers could have helped us better understand the tracheal disease in scleroderma and might have opened new horizons in the field of precision medicine. In fact, new laboratory markers (including TGFβ1, IL-6, sPD-1, sPD-L2, and CXCL4) have been associated with higher mRSS score and more pronounced changes in thoracic CT [3]. Despite these limitations, the quantitative analysis of the trachea through computer software offers a discriminant method that can help produce an objective measure and obtain prognostic information in scleroderma.

In conclusion, the present study shows that, in a sample composed predominantly of scleroderma patients with associated ILD, there were abnormalities in the geometry of the trachea in eccentricity, diameter, and tortuosity. In these patients, abnormalities in the geometry of the trachea were associated with markers of functional obstruction at the level of the cervical trachea. In addition, the measurement of tracheal tortuosity was correlated with cutaneous involvement, the degree of pulmonary fibrosis, and the presence of ATA. Although the encouraging data presented here require further validation in prospective studies, we believe that skeletonization and CT trachea volumetry may improve the ability of radiologists and rheumatologists to accurately assess the tracheas of ​​patients with scleroderma both in clinical practice and in trials.
